# Serum P-Cresyl Sulfate Is Associated with Peripheral Arterial Stiffness in Chronic Hemodialysis Patients

**DOI:** 10.3390/diagnostics15182353

**Published:** 2025-09-16

**Authors:** Yahn-Bor Chern, Chih-Hsien Wang, Chin-Hung Liu, Hung-Hsiang Liou, Jen-Pi Tsai, Bang-Gee Hsu

**Affiliations:** 1 Division of Nephrology, Department of Medicine, St. Michael’s Hospital, Unity Health Toronto, University of Toronto, Toronto, ON M5B 1W8, Canada; 2 Division of Nephrology, Hualien Tzu Chi Hospital, Buddhist Tzu Chi Medical Foundation, Hualien 97004, Taiwan; 3 School of Medicine, Tzu Chi University, Hualien 97004, Taiwan; 4 Graduate Institute of Clinical Pharmacy, School of Medicine, Tzu Chi University, Hualien 97004, Taiwan; 5 School of Pharmacy, Tzu Chi University, Hualien 97004, Taiwan; 6 Division of Nephrology, Department of Internal Medicine, Hsin-Jen Hospital, New Taipei City 24243, Taiwan; 7 Division of Nephrology, Department of Internal Medicine, Dalin Tzu Chi Hospital, Buddhist Tzu Chi Medical Foundation, Chiayi 62247, Taiwan

**Keywords:** cardio-ankle vascular index, peripheral arterial stiffness, C-reactive protein, *p*-Cresyl sulfate, hemodialysis

## Abstract

**Background/Objectives**: Arterial stiffness is a major cardiovascular risk factor in patients with hemodialysis (HD). We conducted a cross-sectional study aimed at determining the relationship between serum p-Cresyl sulfate (PCS) and peripheral arterial stiffness (PAS), defined via the cardio-ankle vascular index (CAVI), in 110 patients receiving chronic HD. **Methods**: Participants were divided into PAS (CAVI ≥ 9.0) and control (CAVI < 9.0) groups. Serum PCS level was measured by high-performance liquid chromatography-mass spectrometry. **Results**: PAS was detected in 37 (33.6%) patients. The PAS patients were older and had higher SBP, more diabetes, and higher serum PCS and C-reactive protein (CRP) than the control group. Upon multivariate analysis, PAS was significantly associated with PCS (adjusted odds ratio: 1.238 per 1 mg/L increase, 95% confidence interval [CI]: 1.119–1.371, *p* < 0.001). The CAVI, advanced age, and CRP demonstrated a significant correlation with PCS, as evidenced by the correlation analysis conducted. Area under the receiver operating characteristic curve analysis showed that PCS had a good diagnostic value for PAS (AUC: 0.872, 95% CI: 0.805–0.939; *p* < 0.001), and the optimal cutoff value was 24.29 mg/L. **Conclusions**: PCS demonstrates great potential as a biomarker in the diagnosis of arterial stiffness.

## 1. Introduction

Chronic kidney disease (CKD) remains a significant global health challenge despite advances in current medical care [1]. Cardiovascular (CV) disease risk is markedly associated with decreased renal function, leading to substantial morbidity and mortality in patients with CKD undergoing maintenance hemodialysis (HD) [2,3,4]. In addition to traditional CV risk factors, nontraditional risk factors, such as inflammation, uremic toxins, chronic volume overload, anemia, and vascular calcification, have received increasing attention for their roles in accelerating vascular damage in this vulnerable population [4,5]. Therefore, there is a heightened emphasis on identifying and understanding the mechanisms through which these nontraditional risk factors contribute to adverse CV events in the setting of CKD, with a focus on their potential as routine diagnostic tools for early identification of subclinical diseases in patients with CKD.

Arterial stiffness (AS) represents a complex and multifaceted condition, referring to a loss of vessel wall elastic compliance and stiffening of arterial walls, and it has been emerging as a CKD-related consequence and a predictor of future development of CV disease and all-cause mortality in CKD, especially end-stage renal disease (ESRD) [6,7,8]. The cardio-ankle vascular index (CAVI), derived from the conventional diagnostic methodology of pulse wave velocity (PWV), serves as a parameter for the assessment of AS, as it evaluates the entire arterial tree from the aorta to the ankle and aims at eliminating the blood pressure (BP) effects seen in PWV exams [9]. Studies showed that CAVI serves as a predictive factor of major CV events and mortality in various populations, making it a clinically valuable tool to assess AS in patients with ESRD, because it can bypass the effect of fluctuating BP values commonly seen in this population [10,11,12,13].

*p*-Cresyl sulfate (PCS) is a protein-bound uremic toxin that is generated during tyrosine and phenylalanine metabolism by gut bacteria [14]. The vascular damage caused by PCS includes oxidative stress, inflammatory response, calcification of arteries, and endothelial dysfunction, all of which contribute to the pathogenesis of AS [15,16]. Although the association of PCS with brachial-ankle PWV (baPWV) or carotid–femoral PWV (cfPWV) has been reported in individuals with CKD, the specific relationship between PCS and peripheral AS (PAS) measured by CAVI to reduce the influence of BP in patients under HD remains an area of ongoing investigation [17,18]. The aims of this study were to investigate the association of serum PCS levels with AS assessed by CAVI, verify whether PCS is independently associated with high PAS, and evaluate its potential as a biomarker to identify high CV risk in patients undergoing HD.

## 2. Materials and Methods

### 2.1. Research Design and Patient Enrollment

The present investigation obtained authorization from the research ethics committee of Tzu Chi Hospital in Hualien, which is affiliated with the Buddhist Tzu Chi Medical Foundation (IRB108-219-A and ratified on 19 November 2019). We performed consecutive sampling of all eligible HD patients at our center during the study period. According to the power analysis, which aimed to identify a correlation coefficient of 0.3 between PCS concentrations and CAVI, utilizing an alpha error threshold of 0.05 and 80% power, it was determined that a minimum sample size of 85 participants was necessary [19]. Patients who were in the HD program of a medical center in Hualien, Taiwan, were recruited between 1 June 2022 and 31 August 2022. The inclusion criteria were age ≥20 years and on regular HD (three times a week) with standard bicarbonate dialysate for >3 months. All patients underwent dialysis using the same high-flux polysulfone disposable artificial kidney (FX class dialyzer; Fresenius Medical Care, Bad Homburg, Germany). There were 242 HD patients who were initially evaluated. We excluded from the study patients who were actively infected (*n* = 6); had undergone amputation (*n* = 12); had malignancy (*n* = 16); had suffered stroke (*n* = 10), peripheral arterial occlusive disease (*n* = 12), or acute heart failure (*n* = 1); those who were bedridden (*n* = 12) at the time of sampling; those who used something other than a high-flux dialyzer (*n* = 33); and those who were unable to understand the study details and provide informed consent (*n* = 30). After applying these exclusion criteria, approximately 110 chronic HD patients were included in the final analysis. Using standard mercury sphygmomanometers with cuff sizes adapted to the participants, trained staff measured resting morning blood pressure (BP) after at least 10 min of rest on Wednesday or Thursday of non-dialysis days. Systolic BP (SBP) and diastolic BP (DBP), corresponding to the beginning and cessation of Korotkoff sounds, respectively, were taken three times at an interval of 5 min; the mean values were recorded. In the prevalence survey, hypertension was operationally characterized as a SBP of 140 mmHg or greater, DBP of 90 mmHg or greater, or the consumption of antihypertensive medications within the preceding two weeks, based on JNC 8 criteria. Diabetes mellitus was confirmed in patients with fasting serum glucose concentrations of not less than 126 mg/dL or those receiving antidiabetic medications.

### 2.2. Anthropometric Data Collection

In a state of minimal attire and without footwear, the subject’s body mass was quantified to the nearest 0.5 kg both before and after a hemodialysis session. Height was recorded to the nearest 0.5 cm. Waist circumference was assessed with precision to the nearest 0.5 cm at the narrowest point located between the inferior margin of the ribs and the iliac crest. The body mass index (BMI) was obtained by dividing the weight in kilograms by the square of the height in meters. All data were collected by a single operator.

### 2.3. Biochemical Determinations

A 5 mL blood specimen was procured from all patients prior to the HD sessions. Kinetic modeling of single-pool urea kinetics was used to calculate, in the pre- and post-dialysis sessions, the urea volumetric kinetics parameter, the fractional clearance index urea (Kt/V), and the urea reduction ratio, respectively. Using 0.5 mL blood samples, blood cell count, such as white blood cell and hemoglobin, was measured by Sysmex SP-1000i (Sysmex America, Mundelein, IL, USA), and the remaining blood was centrifuged at 3000× *g* for 10 min. We separated serum samples within one hour of collection, then refrigerated them at 4 °C and employed them for biochemical tests within one hour of sample collection. Using an autoanalyzer (Siemens Advia 1800; Siemens Healthcare GmbH, Henkestr, Erlangen, Germany), the following serum parameters were measured: albumin, uric acid, glucose, blood urea nitrogen, creatinine, total calcium, phosphorus, total cholesterol, triglyceride, and C-reactive protein (CRP). Serum intact parathyroid hormone concentration was assayed by a commercial enzyme-linked immunosorbent assay kit (Abcam, Cambridge, MA, USA).

### 2.4. Carotid-Ankle Vascular Index Measurement

CAVI was assessed with a waveform device (VaSera VS-1000, Fukuda Denshi, Tokyo, Japan), as per the recommendation. All of the participants underwent a supine position and 10 min rest, with continuous measurements of electrocardiogram and phonocardiogram, BP from the arms and ankles, and an automatic measurement of PWV and CAVI [20]. CAVI measures regional arterial stiffness based on heart-to-ankle PWV (the time from the aortic valve to the ankle), measured in combination with brachial BP [20]. The underlying mathematics are derived from the Bramwell–Hill relationship between PWV and arterial volume change, and ultimately from the stiffness parameter β, as reported by Hayashi et al. [21]. Detailed calculation procedure of CAVI is reported by Miyoshi et al. [20].

In our present study, we apply the proposed expert consensus of the Physiological Diagnosis Criteria for Vascular Failure Committee of the Japan Society for Vascular Failure with a cutoff value of CAVI ≥ 9.0 for diagnosing abnormal vascular function [22]. Therefore, patients with CAVI ≥ 9.0 were classified into the PAS group, and patients with CAVI < 9.0 were classified into the normal CAVI/control group. High CAVI was deemed to represent the presence of PAS.

### 2.5. Quantification of Serum *p*-Cresyl Sulfate by High Performance Liquid Chromatography Mass Spectrometry (HPLC-MS)-Based Measurement

Samples were taken in a pre-dialysis manner and measured with an HPLC-MS (Waters e2695, ACQUITY QDa, Waters Corporation, Milford, MA, USA) [17,18]. Luna^®^ C18 (2) (5 µ, 250 × 4.60 mm, 100 Å) from Phenomenex was the analytical column used, and specific chromatographic conditions were optimized: column temperature at 40 °C, flow rate of 0.8 mL/min, and 30 µL injection volume. An initial binary mobile phase gradient was configured, comprising 95% aqueous solution containing 0.1% formic acid (A) and 5% methanol with 0.1% formic acid (B), which was sustained for 1 min. Thereafter, solvent B was incrementally increased in a linear fashion over a 12 min period to reach a composition of 70%, which was then maintained for an additional 2 min. For column equilibration, solvent B was decreased to 50% within one minute and held for two minutes. The liquid chromatography-mass spectrometry (LC-MS) gradient condition was adjusted to simultaneously measure pretreated samples in positive- or negative-ion (i.e., PCS) mode using electrospray ionization. The parameters used throughout the instruments included a 600 °C desolvation temperature, a 0.8-kV capillary voltage, and a 15.0 V sample cone. The mass spectrometer was operated in full scan mode, covering a mass-to-charge ratio (*m*/*z*) range of 50 to 450 *m*/*z* and 100–350 *m*/*z* in positive and negative-ion modes, respectively. The individual mass of each compound was monitored using single ion recording mode (PCS: 187.0 *m*/*z*). Data collection and processing were performed utilizing Empower^®^ 3.0 software (Waters Corporation, Milford, MA, USA). The retention time of PCS was about 16.56 min. The amount of endogenous substances was determined by analyzing peak areas and comparing them with a calibration curve constructed from standard solutions. All the linearity determination coefficients (r^2^) were >0.995. Single ion analysis was performed using the LC–MS single ion recording mode.

### 2.6. Statistical Analysis

For normal distribution of continuous variables, the Kolmogorov–Smirnov test was used. Continuous variables with normal distribution were represented as mean ± standard deviation and were subjected to comparison between the two groups employing a two-tailed independent Student’s *t*-test. Variables characterized by non-normal distributions were presented as medians and interquartile ranges and were compared across groups using the Mann–Whitney *U* test. Prior to subsequent linear regression analysis, these non-normally distributed data underwent logarithmic transformation. Qualitative variables were expressed as numbers (percentages) and analyzed by the χ^2^ test. Potential risk factors for PAS were determined based on the results of multivariate logistic regression analysis.

Correlations of clinical variables with CAVI (left and right) and log-PCS were examined using Spearman’s correlation coefficient. The receiver operating characteristic (ROC) curve analysis was employed to estimate the area under the ROC curve (AUC) for the optimal PCS cut-off value in predicting PAS in patients on HD (MedCalc Software Ltd., version 22.019, Ostend, Belgium). These were analyzed with the SPSS program for Windows (version 19.0, IBM Corp., Armonk, NY, USA). Statistical significance was considered at *p*-value < 0.05.

## 3. Results

Of 110 patients on maintenance HD included in this study, 37 (33.6%) were in the PAS group, and 73 (66.4%) were in the control group. As shown in Table 1, compared with the control group, the PAS group was significantly older (63.8 ± 9.6 vs. 58.8 ± 11.6 years, *p* = 0.025), had significantly higher SBP (156.8 ± 25.1 vs. 145.9 ± 21.2 mmHg, *p* = 0.018), and comprised significantly higher proportions of diabetes mellitus (64.9% vs. 43.8%, *p* = 0.037) and hypertension (73.0% vs. 53.4%, *p* = 0.048). No statistically significant differences were observed between the two cohorts regarding hemodialysis vintage, body weight, BMI, DBP, hemoglobin, albumin, lipid profile, glucose, urea reduction rate, Kt/V, and nutritional markers (all *p* > 0.05). Compared with the control group, the PAS group had significantly higher serum levels of total PCS [0.41 (0.24–0.87) vs. 0.29 (0.13–0.55) mg/dL, *p* < 0.001] and CRP (31.61 ± 12.52 vs. 16.68 ± 8.03 mg/L, *p* = 0.007).

On multivariate logistic regression (Table 2), every 1 mg/L increase in PCS was associated with a 17.8% increase in the odds of PAS (odds ratio [OR]: 1.178, 95% confidence interval [CI]: 1.106–1.255, *p* < 0.001). Model 1 adjusted for a core set of demographic and major clinical confounders known to influence cardiovascular health, including age, HD vintage, diabetes, hypertension, sex, BMI, SBP, and DBP. After adjustment for these potential confounders, PCS remained independently associated with PAS (adjusted OR [aOR]: 1.199, 95% CI: 1.112–1.292, *p* < 0.001). Model 2 provided the most comprehensive adjustment by including all variables from Model 1 plus a block of key laboratory and dialysis-related parameters pertinent to the uremic milieu. This included markers such as hemoglobin, albumin, lipid, glucose, calcium, phosphorus, blood urea nitrogen, creatinine, intact parathyroid hormone, CRP, initial predialysis urea reduction rate, and Kt/V. Additional adjustment for laboratory levels further increased this association (aOR: 1.238, 95% CI: 1.119–1.371, *p* < 0.001).

Spearman correlation analysis demonstrated that serum PCS level correlated moderately with CAVI on the left (*r* = 0.557) and right (*r* = 0.555) (Table 3, both *p* < 0.001) and that the left and right CAVI were highly intercorrelated (*r* = 0.934, *p* < 0.001). In addition, PCS showed significant albeit weaker positive correlations with age (*r* = 0.244, *p* = 0.010) and log-transformed CRP (*r* = 0.277, *p* = 0.003) but not with vintage of HD, lipid profile, glycemic levels, mineral metabolites, and dialysis adequacy indices. These results may reflect an interrelation among aging-related mechanisms, inflammation, and the burden or action of PCS.

**Figure 1:**
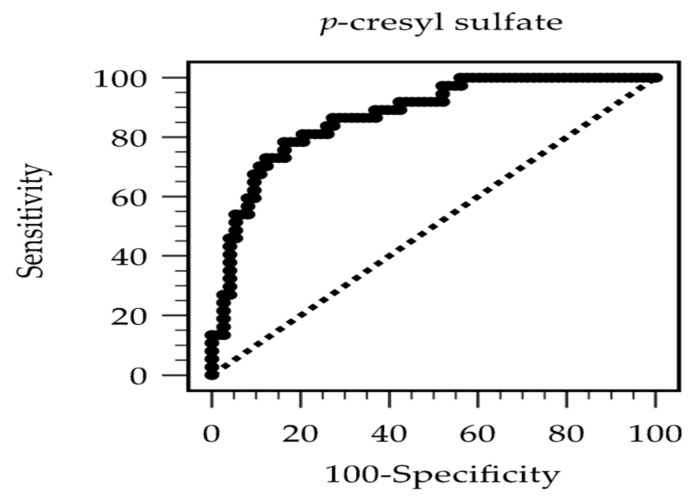
The region encompassed by the receiver operating characteristic curve serves as a metric for the diagnostic efficacy of serum *p*-Cresyl sulfate levels in forecasting peripheral arterial stiffness in individuals undergoing hemodialysis.

The diagnostic ability of PCS to detect PAS was analyzed by the ROC curve. As shown in Figure 1, the AUC was 0.872 (95% CI: 0.805–0.939; *p* < 0.001). The best cutoff serum total PCS was 24.29 mg/L, with 78.38% sensitivity, 83.56% specificity, 70.73% positive predictive value, and 88.40% negative predictive value for PAS diagnosis. The high negative predictive value implied that a PCS level <24.29 mg/L can be particularly useful for excluding PAS in patients on HD.

## 4. Discussion

In this cross-sectional study on patients on maintenance HD, we found a significant association between elevated serum PCS levels and PAS, which was defined by CAVI ≥ 9.0. PCS concentrations were markedly higher in patients with PAS than in those without PAS, even after comprehensive multivariate adjustment for a wide range of demographic, clinical, and laboratory confounders. Notably, serum PCS had strong discriminatory power for PAS, with each 1 mg/L increase in PCS associated with 23.8% higher odds of PAS. These findings suggested PCS as a valuable biomarker for identifying PAS in this high-risk population.

The findings of our study underscore the significance of traditional CV risk factors in the pathology of PAS. First, the relatively high prevalence of PAS in patients with diabetes mellitus was probably the result of hyperglycemia, which can cause oxidative stress, chronic low-grade inflammation, metabolic inflexibility, and endothelial dysfunction, leading to arterial remodeling [23,24]. Second, the expected and markedly higher SBP levels in the PAS group than in the control group likely reflected the continued pressure increase posing chronic shear stress, followed by endothelial dysfunction and vascular remodeling [25]. Third, the relatively high risk of PAS in older patients on HD, which was consistent with previous findings, suggested aggravated AS secondary to altered mechanical properties of the arterial wall with aging (i.e., degradation of elastin and increased collagen deposition) and reactive oxygen species (ROS)-induced inflammation [26,27]. Lastly, the high CRP in patients with PAS indicated the possible role of inflammation in endothelial damage and worsening AS [28].

The primary finding of this study was the strong independent relationship of serum PCS level with the presence of PAS, even after extensive multivariate correction. This highlighted PCS as a marker and potential pathogenic mediator of AS. Mechanistically, multiple pathways are likely involved, as PCS is a well-established vasculotoxin. It has been shown to induce oxidative stress, thereby promoting endothelial dysfunction as well as calcification and inflammation of vascular smooth muscle cells [17,29,30]. In particular, PCS can generate intracellular ROS via nicotinamide adenine dinucleotide phosphate oxidase, resulting in activation of proinflammatory signaling pathways, such as the mitogen-activated protein kinase and nuclear factor kappa-light-chain-enhancer of activated B cells pathways, and consequently an osteogenic vascular cell [15,17]. Our correlation analysis added more clinical content to the role of these pathways. The substantial positive correlation between PCS and CRP was likely to represent a vicious cycle, with PCS accumulating systemic inflammation, and the preexisting inflammatory state of uremia amplifying the vasculotoxic effects of PCS. In addition, despite previous findings of age-related increases in PCS levels in healthy people [31,32], the exact mechanisms underlying the association between age, PCS accumulation, and subsequent pathological outcomes are not well established. The association of age with PCS in this study could be potentially explained by the so-called hypothetical mechanism of “inflamm-aging,” given that older patients had a background that comprised chronic low-grade inflammation and gut microbiota changes, which could favor the production of p-Cresol, a precursor of PCS [33,34,35].

The importance of the findings of this study can be understood in comparison with prior research (Table 4). Previous studies have associated PCS with AS, but none have investigated the extent of that association and diagnostic accuracy. For example, the study by Lai et al. in 2019 applied cfPWV, which is the gold standard measure for central AS, to compare HD groups and found a significant albeit less strong association with PCS, with an AUC of 0.661 (cutoff 18.99 mg/L) [17]. In 2022, Chang et al. found an independent association between baPWV and PCS in a nondialysis CKD population, although the AUC was lower at 0.628 (cutoff 20.49 mg/L) [18]. In this study, we were particularly impressed with the markedly improved AUC of 0.872, which likely reflected two significant methodological differences. First was the selection of a common HD patient population situated at the terminal phase of the uremic spectrum, at which point the deleterious effects of toxin-induced vascular damage were most pronounced. Second and even more critical was the use of CAVI as the measurement modality for PAS. Unlike cfPWV and baPWV, CAVI is designed to be largely independent of the influence of BP during measurement shown in several studies [36,37,38,39]. CAVI also offers practical advantages over PWV, being easier to perform and less time-consuming, which is particularly beneficial in busy HD-unit workflows [20]. Indeed, given the marked hemodynamic instability and BP swings during HD, a BP-independent parameter, such as CAVI, could represent the actual endogenous biological linkage between uremic toxins and arterial wall integrity. This linkage could be partially confounded by BP variability when assessing PWV using the traditional measures.

This study has several limitations. The most important is its cross-sectional nature, which means it can only demonstrate association and not causation. Although it is reasonable to suggest that elevated PCS causes PAS, advanced vascular disease being a cause of increased PCS levels cannot be concluded with absolute certainty. Another limitation of our study is the exclusion of the most critically ill patients, for whom dialysis may not be the best treatment option. Considering the single-center design with defined exclusion criteria, our results might have been influenced by selection and survival bias and may not necessarily apply to the entire HD population. Although extensive adjustments were made, the presence of unmeasured residual confounders, such as other uremic toxins and additional nutritional or inflammatory factors, cannot be completely ruled out. Therefore, the emerging focus of future research should be on extensive prospective longitudinal investigations to validate causality and define PCS as a predictive biomarker for AS progression. With respect to the measurement of PCS, mass spectrometry is not universally available, which limits immediate routine adoption. Broader clinical use would likely depend on centralized testing and assay standardization (calibration, quality control, inter-laboratory comparability, and reference intervals). In the end, only randomized controlled trials can settle the issue of whether interventions targeted at reducing PCS load (i.e., enhanced dialytic strategies and gut-targeted therapies) will decrease the incidence of AS and finally translate into hard CV events. Translating our observations into a clinical setting and decreasing the high CV risk related to this uremic toxin are important.

## 5. Conclusions

Our findings demonstrated a robust and independent association between elevated serum PCS levels and increased PAS among patients on maintenance HD. Notably, a PCS threshold of 24.29 mg/L exhibited good discriminative performance for identifying PAS. Future prospective cohort studies and interventional trials targeting PCS reduction are warranted to determine causality and explore the potential therapeutic relevance of PCS in mitigating vascular stiffness in the HD population.

## Figures and Tables

**Table 1:** Clinical parameters of the 110 participants exhibiting arterial stiffness who were on hemodialysis (CAVI ≥ 9.0) and of those in the control group (CAVI < 9.0).

Characteristics	All Patients (*n* = 110)	Control Group (*n* = 73)	PAS Group (*n* = 37)	*p* Value
Age (years)	60.47 ± 11.16	58.78 ± 11.56	63.81 ± 9.61	0.025 *
HD vintage (months)	65.70 (30.81–101.85)	67.80 (31.50–108.54)	53.88 (27.96–86.10)	0.374
Pre-HD body weight (Kg)	70.73 ± 16.95	70.16 ± 17.68	71.84 ± 15.57	0.625
Post-HD body weight (Kg)	68.50 ± 16.23	68.03 ± 17.01	69.42 ± 14.75	0.672
Body mass index (Kg/m^2^)	26.63 ± 5.27	26.70 ± 5.32	26.50 ± 5.25	0.850
Left CAVI	7.59 ± 2.71	6.06 ± 1.73	10.59 ± 1.52	<0.001 *
Right CAVI	7.56 ± 2.70	6.01 ± 1.71	10.62 ± 1.30	<0.001 *
Systolic blood pressure (mmHg)	149.52 ± 27.04	145.86 ± 21.17	156.78 ± 25.10	0.018 *
Diastolic blood pressure (mmHg)	79.84 ± 12.98	78.73 ± 12.72	82.03 ± 13.39	0.209
White blood cell (×10^3^ μL)	6.51 ± 1.80	6.55 ± 1.79	6.41 ± 1.83	0.706
Hemoglobin (g/dL)	10.72 ± 1.27	10.72 ± 1.45	10.74 ± 0.83	0.926
Albumin (mg/dL)	4.34 ± 0.54	4.39 ± 0.52	4.24 ± 0.57	0.153
Total cholesterol (mg/dL)	150.14 ± 44.58	149.58 ± 46.41	151.24 ± 41.34	0.854
Triglyceride (mg/dL)	147.50 (93.75–220.25)	148.00 (95.00–122.50)	147.00 (90.00–205.50)	0.569
Glucose (mg/dL)	153.50 (114.00–209.50)	148.00 (95.00–222.50)	144.00 (113.00–232.00)	0.952
Blood urea nitrogen (mg/dL)	64.96 ± 17.69	65.82 ± 18.67	63.27 ± 15.68	0.477
Creatinine (mg/dL)	10.34 ± 2.67	10.42 ± 2.63	10.17 ± 2.76	0.650
Total calcium (mg/dL)	9.27 ± 0.91	9.25 ± 0.80	9.32 ± 1.11	0.711
Phosphorus (mg/dL)	4.76 ± 1.35	4.67 ± 1.38	4.94 ± 1.29	0.331
iPTH (pg/mL)	329.55 (203.33–512.25)	334.30 (222.95–508.95)	315.50 (195.70–601.10)	0.922
Uric acid (mg/dL)	6.66 ± 1.51	6.76 ± 1.57	6.46 ± 1.40	0.336
Total *p*-Cresyl sulfate (mg/L)	21.70 ± 12.02	16.68 ± 8.03	31.61 ± 12.52	<0.001 *
C-reactive protein (mg/dL)	0.31 (0.15–0.57)	0.29 (0.13–0.55)	0.41 (0.24–0.87)	0.007 *
Urea reduction rate	0.72 ± 0.05	0.72 ± 0.05	0.72 ± 0.05	0.961
Kt/V (Gotch)	1.28 ± 0.20	1.28 ± 0.19	1.28 ± 0.21	0.994
Female, *n* (%)	50 (45.50)	33 (45.20)	17 (45.90)	0.941
Diabetes mellitus, *n* (%)	56 (50.90)	32 (43.80)	24 (64.90)	0.037 *
Hypertension, *n* (%)	66 (60.00)	39 (53.40)	27 (73.00)	0.048 *
Causes of uremia				
Diabetic nephropathy, *n* (%)	56 (50.90)	37 (50.70)	19 (51.40)	0.947
Glomerulonephritis, *n* (%)	31 (28.20)	23 (31.50)	8 (21.60)	0.276
Hypertensive nephrosclerosis, *n* (%)	18 (16.40)	11 (15.10)	7 (18.90)	0.606

Normally distributed continuous variables are reported as mean ± standard deviation, with values having been compared using Student’s *t*-test; non-normally distributed continuous variables are reported as median values (interquartile range), compared using the Mann–Whitney *U* test; and categorical variables are reported as number (%), compared using the chi-square test. HD, hemodialysis; CAVI, cardio-ankle vascular index; Kt/V, urea’s fractional clearance index; iPTH, intact parathyroid hormone. * *p* < 0.05 was deemed statistically significant.

**Table 2:** Multivariate logistic regression analysis of serum *p*-Cresyl sulfate concentrations in relation to peripheral arterial stiffness in a cohort of 110 individuals undergoing hemodialysis.

*p*-Cresyl Sulfate	Unadjusted		Model 1		Model 2	
	OR (95% CI)	*p* Value	aOR (95% CI)	*p* Value	aOR (95% CI)	*p* Value
Per 1 mg/L increase	1.178 (1.106–1.255)	<0.001 *	1.199 (1.112–1.292)	<0.001 *	1.238 (1.119–1.371)	<0.001 *

Model 1 is adjusted for age, hemodialysis vintage, diabetes mellitus, hypertension, gender, body mass index, systolic blood pressure, and diastolic blood pressure. Model 2 is adjusted for the Model 1 variable and other variables, such as albumin, hemoglobin, total cholesterol, triglyceride, glucose, calcium, phosphorus, blood urea nitrogen, creatinine, intact parathyroid hormone, C-reactive protein, urea reduction rate, and Kt/V. OR, odds ratio; aOR, adjusted odds ratio; CI, confidence interval; Kt/V, fractional clearance index for urea. * A *p*-value of less than 0.05 was deemed to be statistically significant.

**Table 3:** The Spearman correlation coefficients pertaining to the left CAVI, right CAVI, PCS, and an array of clinical variables in individuals receiving hemodialysis.

Variables	Left CAVI		Right CAVI		PCS (mg/L)	
	Spearman Correlation Coefficient	*p* Value	Spearman Correlation Coefficient	*p* Value	Spearman Correlation Coefficient	*p* Value
Left CAVI	—	—	0.934	<0.001 *	0.557	<0.001 *
Right CAVI	0.934	<0.001 *	—	—	0.555	<0.001 *
PCS (mg/L)	0.557	<0.001 *	0.555	<0.001 *	—	—
Age (years)	0.219	0.022 *	0.206	0.031 *	0.244	0.010 *
Log-HD vintage (months)	−0.056	0.561	−0.031	0.748	0.001	0.989
SBP (mmHg)	0.197	0.039 *	0.180	0.059	0.085	0.376
DBP (mmHg)	0.081	0.401	0.060	0.532	0.068	0.480
White blood cell (×10^3^ μL)	−0.038	0.692	−0.062	0.523	0.071	0.462
Hemoglobin (g/dL)	−0.031	0.749	−0.114	0.238	−0.039	0.686
Albumin (mg/dL)	−0.123	0.199	−0.129	0.180	−0.141	0.142
Total cholesterol (mg/dL)	0.076	0.430	0.042	0.662	−0.076	0.431
Log-Triglyceride (mg/dL)	−0.017	0.857	−0.045	0.644	−0.104	0.279
Log-Glucose (mg/dL)	0.039	0.684	0.019	0.840	−0.088	0.363
BUN (mg/dL)	−0.007	0.939	−0.021	0.826	0.029	0.762
Creatinine (mg/dL)	−0.020	0.837	−0.074	0.441	−0.047	0.625
Total calcium (mg/dL)	0.072	0.457	0.089	0.353	−0.102	0.291
Phosphorus (mg/dL)	0.101	0.293	0.014	0.886	0.061	0.525
Log-iPTH (pg/mL)	−0.012	0.900	−0.019	0.845	0.049	0.611
Uric acid (mg/dL)	−0.070	0.464	−0.106	0.270	−0.167	0.080
Log-CRP (mg/dL)	0.205	0.032 *	0.226	0.018 *	0.277	0.003 *
Urea reduction rate	0.113	0.238	0.117	0.224	0.001	0.990
Kt/V (Gotch)	0.118	0.219	0.128	0.184	0.023	0.814

The dataset pertaining to HD vintage, glucose, triglyceride, iPTH, and CRP levels exhibited a non-normal distribution, necessitating a log transformation prior to analytical procedures. The data analysis was conducted utilizing the Spearman correlation analysis. HD, hemodialysis; CAVI, cardio-ankle vascular index; PCS, *p*-cresyl sulfate; SBP, systolic blood pressure; DBP, diastolic blood pressure; iPTH, intact parathyroid hormone; BUN, blood urea nitrogen; CRP, C-reactive protein; Kt/V, fractional clearance index for urea. * *p* < 0.05 was considered statistically significant (two-tailed).

**Table 4:** Comparative analysis of studies investigating *p*-Cresyl sulfate and arterial stiffness.

Study (Author, Year)	Population	N	Arterial Stiffness Measurement	Key Association Metric	AUC (95% CI)	Optimal Cutoff (mg/L)
Current study	HD	110	CAVI	aOR: 1.238 per 1 mg/L increase	0.872 (0.805–0.939)	24.29
Lai et al. (2019) [17]	HD	118	cfPWV	aOR: 1.067 per 1 mg/L increase	0.661 (0.568–0.746)	18.99
Chang et al. (2022) [18]	Non-dialysis CKD (stage 3–5)	160	baPWV	aOR: 1.098 per 1 mg/L increase	0.628 (0.531–0.725)	20.49

CAVI: cardio-ankle vascular index; cfPWV: carotid-femoral pulse wave velocity; baPWV: brachial-ankle pulse wave velocity; CKD: chronic kidney disease; aOR: adjusted odds ratio; AUC: area under the curve.

## Data Availability

The author responsible for correspondence is able to provide the data employed in this research upon request.

## Abbreviations

The following abbreviations are used in this manuscript:

AUC	Area under the curve
baPWV	Brachial-ankle pulse wave velocity
BMI	Body mass index
BP	Blood pressure
CAVI	Cardio-ankle vascular index
cfPWV	Carotid-femoral pulse wave velocity
CKD	Chronic kidney disease
CRP	C-reactive protein
CV	Cardiovascular
DBP	Diastolic blood pressure
ESRD	End-stage renal disease
Kt/V	The fractional clearance of urea, adjusted for the patient’s total body water.
HD	Hemodialysis
LC-MS	Liquid chromatography-mass spectrometry
PAS	Peripheral arterial stiffness
PCS	p-Cresyl sulfate
PWV	Pulse wave velocity
ROC	Receiver operating characteristic
ROS	Reactive oxygen species
SBP	Systolic blood pressure
